# Socioeconomic inequity in extreme outcomes within very pre-term and/or very low birthweight infants: evidence from multi-national cohorts

**DOI:** 10.3389/fpubh.2026.1791450

**Published:** 2026-04-23

**Authors:** Hanifa Pilvar, Catia Nicodemo, Stavros Petrou, Brian A. Darlow, Paula van Dommelen, Kari Anne I. Evensen, Sarah Harris, John Horwood, Samantha Johnson, Neil Marlow, Karen Mathewson, Saroj Saigal, Louis A. Schmidt, Dieter Wolke, Lianne J. Woodward, Sungwook Kim

**Affiliations:** 1 Nuffield Department of Primary Care Health Sciences, University of Oxford, Oxford, United Kingdom; 2 Brunel Business School, Brunel University London, London, United Kingdom; 3 Department of Paediatrics and Child Health, Christchurch University of Otago, Dunedin, New Zealand; 4 The Netherlands Organization for Applied Scientific Research (TNO), The Hague, Netherlands; 5 Department of Clinical and Molecular Medicine, Norwegian University of Science and Technology, Trondheim, Norway; 6 Department of Psychological Medicine, University of Otago, Dunedin, New Zealand; 7 Department of Population Health Sciences, University of Leicester, Leicester, United Kingdom; 8 University College London, London, United Kingdom; 9 Department of Psychology, Neuroscience and Behaviour, McMaster University, Hamilton, ON, Canada; 10 McMaster University, Hamilton, ON, Canada; 11 Department of Psychology, University of Warwick, Coventry, United Kingdom; 12 School of Health Sciences, University of Canterbury, Christchurch, New Zealand

**Keywords:** concentration index, horizontal inequity, inequality, RECAP Pre-term Project, very low birth weight, very pre-term

## Abstract

**Background:**

Pre-term birth (< 37 weeks' gestation) is a major cause of neonatal mortality, with very pre-term (< 32 weeks' gestation) and extremely pre-term (< 28 weeks' gestation) infants facing the highest risks. While socioeconomic disparities in pre-term birth are well-documented, relatively little is known about inequities among the highest risk subgroups.

**Methods:**

Using data from the RECAP Pre-term Project across six high-income countries, we analyzed socioeconomic inequality in the incidence of extreme pre-term birth (EP) and extremely low birth weight (ELBW) among very pre-term/very low birth weight (VP/VLBW) infants. We measured inequality using concentration indices across cohorts and two adjusted measures of horizontal inequity (HI1 and HI2), to estimate the contributions of socioeconomic factors (e.g., parental education, ethnicity) to the outcomes.

**Results:**

Results showed that the incidence of EP in the Netherlands (HI_2_ = 0.171) and Norway (HI_2_ = 0.210) was higher among more socioeconomically advantaged infants born at VP/VLBW, but higher among more socioeconomically disadvantaged infants in New Zealand (HI_2_ = −0.020). Incidence of ELBW was higher among disadvantaged infants in Germany (HI_2_ = −0.046). Parental education was the strongest driver of these effects, though ethnicity and socioeconomic status moderated these effects.

**Conclusions:**

Counterintuitively, EP/ELBW were concentrated among advantaged groups in some countries, possibly reflecting survival bias or unequal access to neonatal care. The study highlights the need for targeted policies addressing inequities within high-risk pre-term populations and underscores methodological challenges in assessing disparities among vulnerable subgroups.

## 1 Background

Pre-term birth, birth occurring before 37 weeks' gestation, represents a critical global health challenge with significant implications for infant survival and development. The World Health Organization (WHO) distinguishes between moderate and late pre-term birth (between 32^+0^ and 36^+6^ weeks), very pre-term birth (before 32 weeks) and extremely pre-term birth (before 28 weeks), with the last subgroup carrying the highest risks of sequelae. Annually, an estimated 10% of newborns worldwide are born pre-maturely (1), making pre-term birth the foremost cause of mortality in children under five. Although the burden is heaviest in Southern Asia and sub-Saharan Africa, no region remains unaffected by this pressing public health concern.

Inequality refers to the observable differences in health outcomes, such as the degree of pre-maturity, across various groups in a given population. Inequity, on the other hand, highlights differences that are deemed unfair or unjust, typically arising from factors that are not related to legitimate differences in need, such as maternal age (2). To isolate the element of unfairness, inequality can be adjusted for legitimate medical needs, resulting in a measure known as horizontal inequity. This measure reflects disparities that cannot be explained by variations in need and provides a clearer understanding of inequity in healthcare access and outcomes (2, 3).

This paper specifically investigates socioeconomic inequality and inequity in Extreme Pre-term (EP; < 28 weeks' gestation) or Extreme Low Birth Weight (ELBW; < 1,000 g weight at birth) within Very Pre-term (VP; < 32 weeks' gestation) and Very Low Birth Weight (VLBW; < 1,500g weight at birth), comparing patterns across multiple high-income countries, while assessing how various socioeconomic factors contribute to observed disparities.

Although the unequal distribution of pre-term birth across socioeconomic groups has been extensively documented (4–8), existing research has typically examined pre-maturity through a binary pre-term/term classification, thereby overlooking important variations within sub-categories of pre-term birth. While the elevated medical needs of infants born at less than 32 gestational weeks compared to term infants are well-established, significant knowledge gaps remain regarding how socioeconomic inequities manifest specifically among very pre-term infants. Understanding these differences is key for identifying the most vulnerable subgroups and tailoring interventions to improve their health and developmental outcomes.

VP birth is relatively rare (approximately 1.5% of all births according to 1) and research studies on these vulnerable infants often lack the appropriate sample size for a robust analysis. However, we have access to data from the RECAP Pre-term Project, a large-scale collaboration of longitudinal cohorts born VP/VLBW in high-income countries, which enables an analysis of pre-term subgroups. Our study includes cohorts from six countries: the Netherlands, Germany, New Zealand, Canada, Norway, and the United Kingdom. These cohorts provided harmonized individual-level data on birth outcomes and socioeconomic background, making it possible to conduct a cross-country comparison of inequity within the VP/VLBW population.

## 2 Method

### 2.1 Study population

Our data came from the RECAP Pre-term Project (9–11), a platform of longitudinal cohort studies of individuals born VP/VLBW in high income countries. For this analysis, we utilized data from six countries: the Netherlands (POPS^1^), Germany (BLS^2^ from Bavaria), New Zealand [NZ, see eg., (12)], Canada (CAN), Norway [NTNU^3^, see eg., (13)], and the United Kingdom [EPICure 1, see e.g., (14)].

Table 1 provides an overview of the birth year for each cohort and the inclusion criteria for each study and indicates whether a term-born control group (GA ≥ 37 weeks) was available for comparison.

**Table 1:** Specification of data sets.

Geographic region	Birth year	Selection criteria	Control group
Bavaria, Germany (BLS)	1985–86	GA < 32 or BW < 1,500 g	Term born
The Netherlands (POPS)	1983	GA < 32 and/or BW < 1,500 g	Not available
New Zealand (NZ)	1986	BW < 1,500 g	Term born
Norway (NTNU)	1986–88	BW < 1,500 g	Term born
**In** **Appendix** **only**			
Canada (CAN)	1977–82	BW < 1,000 g	Term born
UK (EPICure 1)	1995	GA < 26	Term born

GA stands for gestation age and BW stands for birth weight.

The study encompasses six national cohorts with varying birth years. Four cohorts originate from the 1980s (Netherlands, Norway, Germany, New Zealand), while the Canadian cohort is marginally older; children within the Canadian cohort were born during a period spanning the late 1970s and early 1980s which makes them 1–8 years older than the median age of children in the other four cohorts. In contrast, the UK EPICure 1 cohort represents a substantially more recent population, with birth years approximately 10 years later than the median of the four cohorts.

Regarding inclusion criteria, four countries selected participants based on very low birth weight (BW < 1,500 grams) and/or very pre-term birth (GA < 32 weeks) status. In contrast, the selection criteria for the Canadian and the UK EPICure 1 cohorts were more stringent, focusing specifically on extremely low birth weight (BW < 1,000 grams) and extremely pre-term birth (GA < 26 weeks), respectively. Due to these differences in cohort definitions and selection criteria, we presented the results for Canada and the UK only in the Appendix. This decision was made because nearly all participants in the Canadian and UK cohorts were extremely pre-term by design, resulting in no meaningful variability in the EP outcome. Consequently, inequality analyses based on binary extreme pre-maturity measures were not informative for these cohorts, and we instead analyzed continuous measures of gestational age and birth weight for them in the Appendix.

Our analysis was restricted to the VP/VLBW group, comprising very pre-term (VP) and/or very low birth weight (VLBW) infants. While certain cohorts included term-born control groups, these were excluded from our study for two key reasons: (1) our dataset lacked intermediate cases with gestational ages between 32–37 weeks, and (2) we had no records of infants with birth weights between 1,500–2,500 grams (occasionally, we observe some infants with gestation age between 32–37 weeks whose birthweight is below 1,500 g or vice versa). This deliberate selection criterion ensured methodological consistency by maintaining an exclusive focus on the VP/VLBW population for all subsequent analyses.

### 2.2 Outcome variables

Our analysis primarily focused on two key birth outcomes: extreme pre-term birth (EP) EP and extremely low birth weight (ELBW). As almost all observations in the Canadian and UK cohorts were either EP or ELBW, we additionally presented results for two continuous outcomes, gestational age (GA) and birth weight (BW), in the Appendix. GA and BW also serve as sensitivity analyses to assess the robustness of the main results based on the binary EP and ELBW measures for all other countries.

GA is measured in completed weeks of pregnancy and reflects the duration of fetal development from conception to birth. BW is measured in grams. EP and ELBW are binary variables, defined as follows: EP indicates births occurring before 28 weeks of gestation, while ELBW indicates a birth weight of < 1,000 grams.

### 2.3 Inequality variable

To ensure comparability across cohorts, we selected a socioeconomic measure that was available in all six cohorts. As shown in Table A1, parental education was the only consistently observed variable across all cohorts, enabling harmonization and comparisons of data across countries.

We harmonized parental education according to the International Standard Classification of Education (15), categorizing it into three levels. Low educated refers to lower secondary education (ISCED 0–2), mid educated corresponds to upper secondary and post-secondary non-tertiary education (ISCED 3–5), and high educated encompasses tertiary education (ISCED 6–8).

Throughout this paper, we used the term “pro-rich” when inequality or inequity indices were positive and “pro-poor” when they were negative, following conventional terminology. However, it is important to note that by “pro-rich” we meant “pro-advantageous” in terms of parental education, rather than income or wealth.

### 2.4 Medical need and non-need variables

To identify need variables, we followed the guidelines of the US National Institute of Child Health and Human Development (NICHD^4^). These variables capture medical and biological factors that directly influence birth outcomes. In contrast, non-need variables represent socioeconomic and demographic factors that do not directly reflect medical needs but may contribute to inequality.

The analysis incorporated several need variables including maternal age, nulliparity, and risky behaviors such as smoking. Regarding non-need variables, the study examined parental education, socioeconomic status (SES), ethnicity, marital status, and infant sex.

### 2.5 Statistical analysis

To measure socioeconomic inequality within our sample of very pre-term infants, we computed the Concentration Index (CI) using the standard approach. In intuitive terms, the concentration index summarizes whether an outcome is disproportionately concentrated among individuals from more or less advantaged socioeconomic backgrounds. A positive value indicates that the outcome is more common among infants from more highly educated families, whereas a negative value indicates concentration among those from less educated families. A value close to zero suggests little or no systematic socioeconomic gradient. In this way, the index provides a single summary measure of the direction and magnitude of inequality across the full distribution of parental education. More specifically, we define the concentration index as in Equation 1:


$$CI=\frac{2}{y}cov(y_{i}\;,\;R_{i})$$ (1)

where *y*_*i*_ represents the outcome measure (e.g., birth at < 28 weeks i.e. incidence of EP), and *R*_*i*_ is the fractional rank of individual *i* in the distribution of the socioeconomic variable (here, parental education). In this study, we used four measures of birth outcomes: two binary measures, EP or ELBW and two continuous measures, GA (in weeks) or BW (in grams) (continuous measures were analyzed in the Appendix). The socioeconomic variable was parental education, chosen due to its availability across all cohorts, ensuring comparability.

We then decomposed the CI following the methods proposed by (3). The decomposition method employs a regression-based approach to explore the determinants of inequality, identifying the contribution of different health need and non-need factors to the inequity observed in the concentration index. Need factors are medical confounders of the outcome, while common indicators of non-need variation measure socioeconomic characteristics. In this study, need factors include maternal age, nulliparity, and maternal risky behaviors such as smoking during pregnancy. Non-need factors include parental education, parental socioeconomic status index, maternal ethnicity, maternal marital status, and infant's gender.

To decompose the concentration index, we first estimated the partial elasticities using the following regression model (probit regression for binary variables):


$$\begin{array}{l} y_{i}=\alpha +\Sigma_{k}\gamma_{k}x_{ki}+\Sigma_{p}\delta_{p}z_{pi}+u_{i}\; \end{array}$$

where *x*_*k*_ represents the list of need factors, and *z*_*p*_ represents the list of non-need factors. The partial elasticity for each variable is calculated using Equation 2:


$$\eta_{k}=\gamma_{k}\;\frac{{\bar{x}}_{k}}{y}\;,\;\eta_{p}=\delta_{p}\;\frac{{\bar{z}}_{p}}{y}$$ (2)

The Concentration Index is then decomposed using Equation 3:


$$\begin{array}{l} CI=\Sigma_{k}\eta_{k}C_{xk}+\;\Sigma_{p}\;\eta_{p}C_{zp}+GC_{u} \end{array}$$ (3)

where *C*_*xk*_ and *C*_*zp*_ are the concentration indices of need and non-need factors, respectively, η_*k*_ and η_*p*_ are the estimated partial elasticities, and *GC*_*u*_ is the generalized concentration index of the error term (unexplained inequality).

However, not all observed inequality is necessarily unfair. Some variation in health outcomes can be explained by legitimate differences in medical need. To distinguish between fair and unfair sources of inequality, we calculated horizontal inequity, defined as the part of socioeconomic inequality that remains after accounting for differences in clinical risk factors. In other words, horizontal inequity captures unequal outcomes among individuals with the same level of clinical need, and is therefore widely regarded as a measure of inequity in healthcare or health outcomes (2, 3). To assess horizontal inequity, using the conventional method, we estimated Equation 4:


$$\begin{array}{l} HI1=CI-\Sigma_{k}\eta_{k}C_{xk} \end{array}$$ (4)

where *HI*1 > 0 indicates pro-rich horizontal inequity, meaning that higher values of the outcome are concentrated among the socioeconomically advantaged individuals, and *HI*1 < 0 indicates pro-poor horizontal inequity.

In practical terms, horizontal inequity measures whether infants with similar clinical risk profiles experience systematically different outcomes according to socioeconomic background. For example, if two infants have comparable medical need at birth but differ in parental education, horizontal inequity captures whether the infant from the more advantaged family has a systematically different probability of being born extremely pre-term or extremely low birth weight after accounting for need. A positive value indicates that, for the same level of medical need, extreme outcomes are more concentrated among infants from more highly educated families, whereas a negative value indicates concentration among those from less educated families.

We then refined our measure of horizontal inequity by applying the decomposition approach proposed by Van de Poel et al. (16), which improves upon the standard method by distinguishing between two types of need: homogeneous need and corrected need. Homogeneous need assumes that all individuals respond to medical needs in the same way, while corrected need accounts for the fact that individuals from different socioeconomic backgrounds may experience different outcomes even with the same level of medical need. This adjustment helps to avoid underestimating the true level of inequity. We refer to this refined measure as *HI*2, and further details on the model are provided in Appendix.

To estimate the coefficients used in the decomposed concentration index where the outcome variable is binary, a probit model was used (3). A probit model allows the estimation of probabilities or marginal effects while imposing a normal distribution on the data. Confidence intervals for *HI*1 and *HI*2 were calculated using bootstrapping with 1,000 random samples, and all results were reported with the 95% confidence level. All analyses were carried out using R, and the rineq package was used for computing the CI.

## 3 Results

### 3.1 Summary statistics

Table 2 shows the summary statistics of outcome variables, inequality variables, need and non-need variables for the pooled data of all cohorts. Table A1 shows the summary statistics separately for each country. The average gestational age at birth was 29 weeks and ranged from 24.5 weeks in the UK to 30 weeks in the Netherlands. The average birthweight was 1,130 grams and ranged from 747 grams in the UK to 1,248 grams in the Netherlands. On average, 35 and 39% of infants were EP and ELBW, respectively. EP and ELBW varies from 20% in the Netherlands to almost 100% in the UK and Canada. These differences reflect the variation in inclusion criteria and birth characteristics across cohorts.

**Table 2:** Summary statistics of the pooled data.

Variable	*N*	Mean	SD	CI	CI SD	Available cohorts
GA (weeks)	2,808	29.0	3.2	0.005	0.001	All
BW (grams)	2,808	1,131	336	0.015	0.004	All
EP (EP = 1)	2,808	0.351	0.477	−0.115	0.028	All
ELBW (ELBW = 1)	2,808	0.390	0.488	−0.129	0.026	All
Parental education (low = 1)	2,037	0.272	0.445	−1	0.014	All
Parental education (middle = 1)	2,037	0.474	0.499	0.035	0.031	All
Parental education (high = 1)	2,037	0.254	0.435	1	0.013	All
Maternal age (years)	2,729	27.361	5.252	0.023	0.002	All
Nulliparous (nulliparous = 1)	2,490	0.500	0.500	0.024	0.026	BLS, POPS, NZ, CAN, UK
Maternal smoking (smoking = 1)	1,290	0.333	0.471	−0.186	0.037	POPS, CAN
Maternal marital status (single = 1)	1,792	0.117	0.321	−0.189	0.054	BLS, POPS, CAN
Ethnicity (Caucasian = 1)	2,114	0.837	0.370	0.168	0.041	POS, NZ, NTNU, CAN, UK
Infant sex (female = 1)	2,804	0.475	0.499	0.010	0.026	All

Table shows number of observations, mean, standard deviation (SD), concentration index (CI) and standard deviation of CI estimate for each variable listed. Last column specifies the cohorts for which the variable is available. GA is gestational age in years and BW is the birth weight in grams. EP and ELBW are binary variables which take one if the child is extremely pre-term or with extreme low birth weight, respectively and zero otherwise. Parental education has three levels. Low educated refers to lower secondary education (ISCED 0–2), mid educated corresponds to upper secondary and post-secondary non-tertiary education (ISCED 3–5), and high educated encompasses tertiary education (ISCED 6–8). Nulliparous is a binary variable which takes one if the mother is in her first pregnancy and zerp otherwise. Maternal smoking is a binary variable which takes one when the mother reported smoking during the pregnancy and zero otherwise. Marital status is a binary variable which takes one when the mother is not married and not in civil partnership and zero otherwise. Ethnicity is a binary variable which takes one when the mother is Caucasian and zero otherwise. Infant sex is a binary variable which takes one when the child is female and zero otherwise. Separate cohort estimates are available in Table A1. Missing values were excluded from the analysis.

In terms of the inequality variables (Table 2) showed that the proportion of parents with low education was, on average, 27; 42% received middle education and 25% received high education.

Maternal age, measured in years and available for all cohorts, averaged 27 years across the sample. Nulliparity, defined as a binary variable indicating first pregnancy, was recorded in all cohorts except Norway, with approximately 50% of the sample classified as nulliparous. Data on risky behaviors, particularly smoking during pregnancy, were only available for the Dutch and Canadian cohorts, revealing that 33% of mothers in these cohorts reported smoking during pregnancy.

Parental education data, harmonized across all cohorts as described previously, served as a key socioeconomic indicator. Parental SES, represented as a continuous variable where higher values denote higher socioeconomic standing, was available for Germany, Canada, Norway, and New Zealand. However, the SES measures could not be harmonized across countries and were therefore analyzed separately for each nation (see Table A1). Ethnicity information, coded as a binary variable (Caucasian/non-Caucasian), was present for all cohorts except Germany, with 83% of mothers identified as Caucasian. Marital status data, indicating whether mothers were unmarried or not in civil partnerships at the time of giving birth, were, on average, 11.7% and were available for Germany, the Netherlands, and Canada. Infant sex was consistently documented across all cohorts, with females representing 47% of the sample. Complete variable descriptions and detailed distributions are provided in Table 2 and Table A1.

### 3.2 Inequality and inequity indices

**Figure 1:**
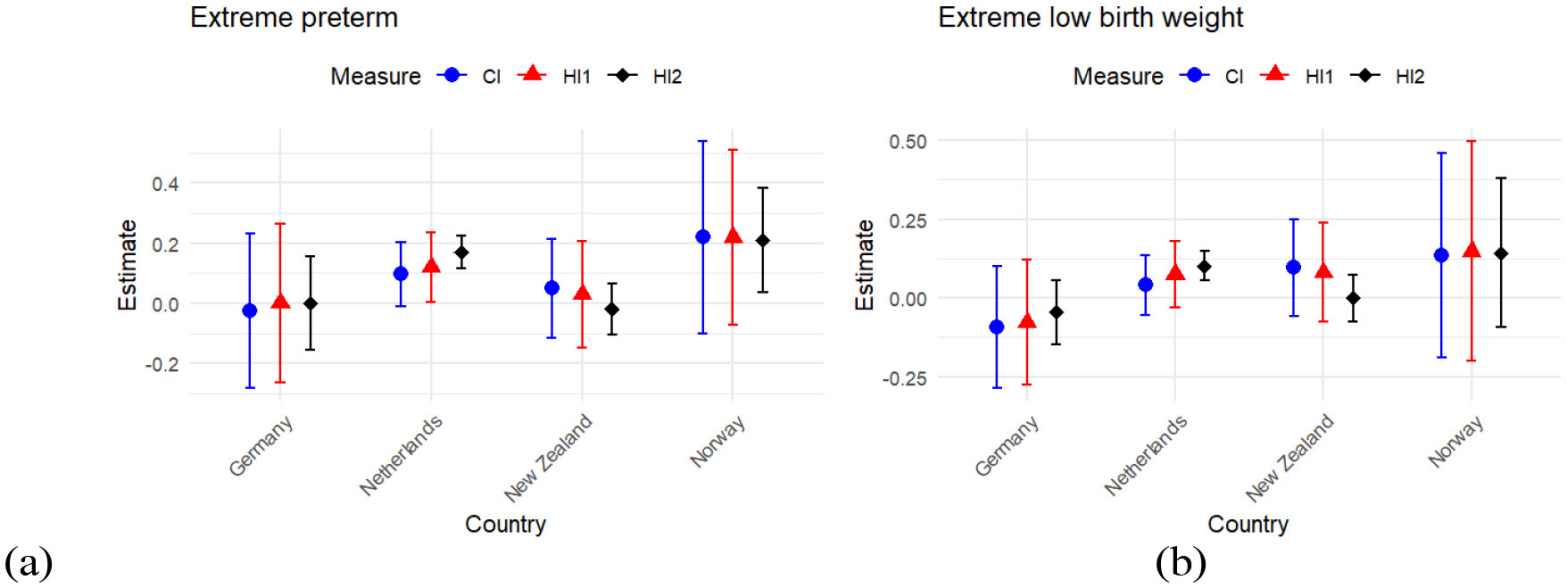
shows the concentration index and inequity indices for birth outcomes. CI (Concentration Index, blue dots) measures the degree of socioeconomic inequality in the outcome across the distribution of parental education. HI1 (red triangles) and HI2 (black diamonds) are measures of horizontal inequity, based on conventional method and Van de Poel et al. (16) method, respectively. They represent the portion of inequality that remains after adjusting for medical need variables. Positive values (pro-rich) indicate that the outcome is concentrated among infants from more highly educated families, while negative values (pro-poor) indicate concentration among infants from less educated families. Values closer to zero indicate little or no socioeconomic gradient.

Figure 1 shows the inequality index (CI) as blue dots, along with two horizontal inequity indices. The first, HI1, represents horizontal inequity using the conventional method and is shown as red triangles. The second, HI2, represents horizontal inequity using the method proposed by Van de Poel et al. (16) and is displayed as black diamonds. These indices are presented for two birth outcomes—EP and ELBW—and are shown separately for the four cohorts included in the study. The figure also includes the associated 95% confidence intervals.

Focusing on HI2 as our main inequity index, we observed that the Netherlands and Norway exhibited strong pro-rich inequity in terms of both EP and ELBW. Specifically, the HI2 index for EP was highest in Norway (0.210), followed by the Netherlands (0.171); these estimates were statistically significant at the 5% level. The HI2 index for ELBW was 0.101 and 0.143 in the Netherlands and Norway, respectively.

On the other hand, Germany and New Zealand exhibited some degree of pro-poor inequity. For example, in terms of EP, New Zealand showed an HI2 index of −0.020, and in terms of ELBW, Germany had an HI2 index of −0.046. For further details and a comparison between CI, HI1, and HI2, see Table A2.

To examine the degree of inequity in Canada and the UK, we refer to Figure A1, which present the CI, HI1 and HI2 results for GA and BW. The HI2 for GA was negative in both countries: −0.003 for Canada and approximately zero (−0.0001) for the UK, indicating a pro-poor inequity—i.e., higher GA among the disadvantaged. This pattern was consistent with findings from Norway and the Netherlands. In contrast, Germany's HI2 for GA was close to zero (0.0002), while New Zealand again showed a different pattern, with a positive HI2 (0.001).

For BW, Canada, New Zealand, and the UK all displayed pro-rich inequity, with HI2 equal to 0.003, 0.006, and 0.004, respectively; whereas Germany, the Netherlands, and Norway showed pro-poor patterns with HI2 equal to −0.008, −0.008, and −0.009, respectively. For further details, see Table A2.

We were unable to present pooled results for our main analysis due to the differing sets of available need and non-need measures across countries. By presenting country-specific results, we were able not only to compare patterns across different national contexts but also to incorporate a broader and more detailed set of need and non-need variables within each cohort. However, we attempted to provide pooled results based on the common need and non-need variables across the cohorts. Specifically, we adjusted for maternal age as a need factor, whereas parental education and infant sex were included as non-need factors. The results were presented in Table A4. There were only slight differences between CI, HI1, and HI2, which can be attributed to the limited number of variables available for adjustment. The results were stronger in terms of value and align with the country-level analysis in terms of direction. EP and ELBW showed indices of 0.245 and 0.201, respectively while GA and BW showed inequity indices of −0.015 and −0.031, respectively.

### 3.3 Contribution of non-need factors

In this subsection, we present the contribution of each non-need variable to inequality, i.e., their contribution to the concentration index (CI). Understanding the relative impact of these non-need variables helps to identify the most influential socioeconomic determinants of disparities in birth outcome. Non-need variables included infant's sex, parental education, parental SES, maternal marital status, and ethnicity.

**Figure 2:**
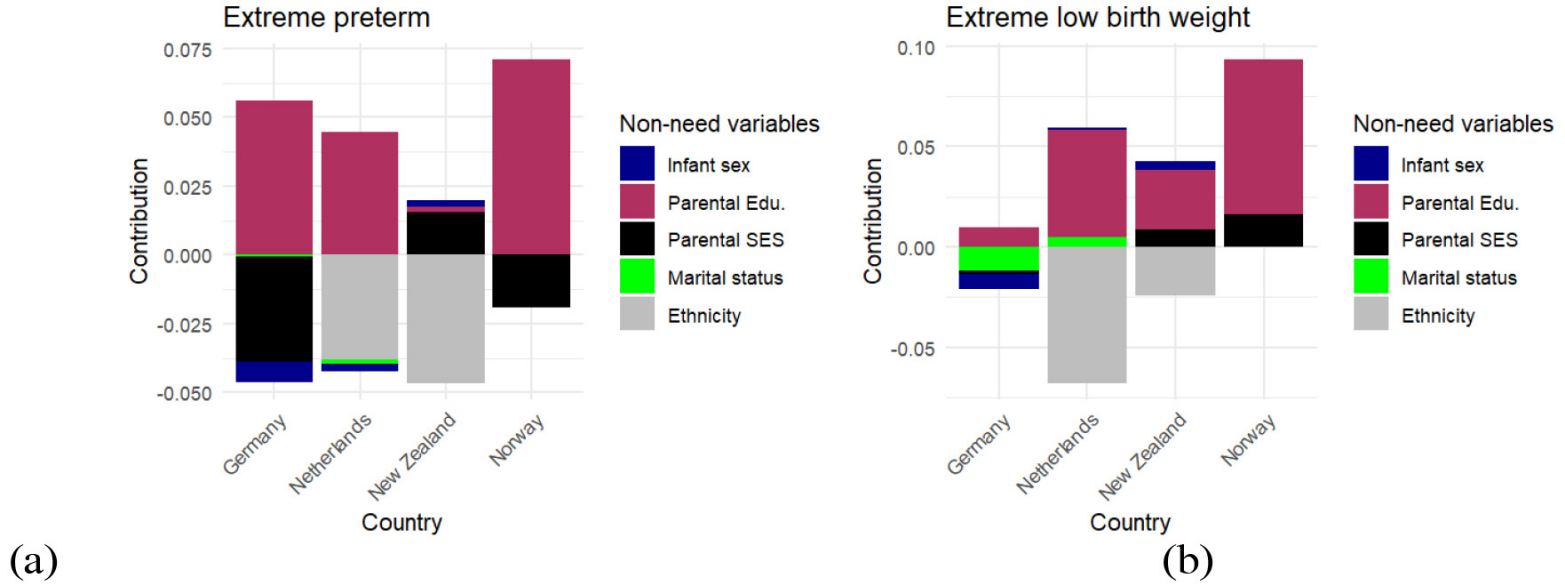
shows the contribution of non-need variables. Infant sex (navy color) is a binary variable which takes one when the child is female. Parental education (maroon color) has three levels. Low educated refers to lower secondary education (ISCED 0-2), mid educated corresponds to upper secondary and post-secondary non-tertiary education (ISCED 3-5), and high educated encompasses tertiary education (ISCED 6-8). Parental SES (black color) is a continuous index of socioeconomic status. Marital status (light green color) is a binary variable which takes one when the mother is not married and not in civil partnership. Ethnicity (grey color) is a binary variable which takes one when the mother is Caucasian.

Figure 2 showed the contribution of these factors separately for each country, allowing for a detailed comparison of how each socioeconomic characteristic influenced inequality across different settings.

The left panel of Figure 2 showed the contribution of non-need variables to inequality in EP. Parental education emerged as a significant contributor, consistently increasing pro-rich inequality, indicating that higher parental education was associated with a higher prevalence of EP. Its contribution was 0.06 in Germany, 0.04 in the Netherlands, 0.002 in New Zealand, and 0.07 in Norway. Ethnicity also played a substantial role in contributing to inequality and, in some cases, surpassed the effect of parental education. This was particularly evident in the Netherlands and New Zealand, with contributions equal to 0.04 and −0.05, respectively. Finally, parental SES also made a noticeable contribution. It contributed to inequality by −0.04 in Germany, 0.02 in New Zealand, and −0.02 in Norway.

It is worth noting that, in some cases, for example in Germany and Norway in terms of EP, parental SES and ethnicity worked in the opposite direction to parental education, decreasing pro-rich inequality. This suggested that socioeconomic advantages related to parental SES and ethnic background mitigated some of the disparities introduced by differences in parental education.

The right-sided panel of Figure 2 shows the contribution of non-need variables to inequality in ELBW. As with EP, parental education remained the most significant contributor, consistently increasing pro-rich inequality. Its contribution was 0.01 in Germany, 0.05 in the Netherlands, 0.03 in New Zealand, and 0.08 in Norway. Ethnicity also played a substantial role, contributing to pro-poor inequality. Its contribution was −0.07 in the Netherlands and −0.02 in New Zealand. Additionally, marital status and infant gender in Germany emerged as important contributors to inequality, indicating that family structure and demographic characteristics also influenced disparities in ELBW outcomes (please see Table A3 for more details).

The left-sided panel of Figure A2 shows the contribution of non-need variables to inequality in GA. Parental education emerged as the main contributor, consistently increasing pro-poor inequality across all countries. This indicated that higher parental education was associated with lower GA, disproportionately affecting disadvantaged groups. Where available, ethnicity and parental SES also played significant roles.

The right-sided panel of Figure A2 shows the contribution of non-need variables to inequality in BW. We observed mixed results regarding the role of parental education. While it remained a significant contributor, it increased pro-poor inequality in Germany, New Zealand, and Norway, indicating that higher parental education in these countries was associated with lower BW. In contrast, parental education had a negligible effect on pro-poor inequality in the Netherlands and even decreased pro-poor inequality in Canada and the UK. This suggested that in the latter two countries, higher parental education was linked to improved BW outcomes, which helped mitigate inequality. These contrasting patterns reflected cross-country differences in how educational advantages translated into birth outcomes, specifically BW.

In the analysis of the pooled data, presented in Table A5, we assessed only the contributions of parental education and infant sex. Parental education was the main contributor, reducing pro-rich inequality for EP, ELBW, and GA, while increasing it for BW.

## 4 Discussion

This study examined socioeconomic inequality and inequity in birth outcomes within a sample of VP/VLBW individuals from six high-income countries: the Netherlands, Germany, New Zealand, Canada, Norway, and the UK. Using harmonized data from the RECAP Pre-term Project, we focused on two key birth outcomes: EP, and ELBW and presented further results for GA and BW in the Appendix. We limited our analysis to individuals born at VP/VLBW, enabling us to study disparities within a high-risk population.

To quantify and decompose inequality, we used the CI and applied the decomposition method by Van de Poel et al. (16), which accounts for both medical need and non-need factors. We observed varying patterns of inequity across countries, with pro-rich inequity in the Netherlands, and Norway, and pro-poor inequity in New Zealand and Germany. The results for Canada and the UK were less consistent, which may reflect the limited variability in GA and BW within these cohorts.

The measures of horizontal inequity for extreme pre-term birth (EP) are 0.210 and 0.171 for the Netherlands and Norway, respectively, while the horizontal inequity measures for extremely low birth weight (ELBW) in the same countries are 0.101 and 0.143, respectively. In terms of magnitude, these values are on the higher end of inequity index estimates for neonatal outcomes reported in other studies (17, 18). Although there are no studies specifically on extreme pre-term birth, one study on low birth weight in general populations reports a concentration index (CI) of −0.05, which is similar to the estimates for New Zealand and Germany (19).

By decomposing the CI, we showed that parental education is the dominant non-need contributor across outcomes, followed by ethnicity and then parental SES. Marital status and infant sex also contribute in specific country contexts. For example, we observe some contribution of infant sex and marital status in the inequity in terms of ELBW in Germany.

Socioeconomic inequities in pre-term birth have been widely documented in the literature, highlighting the persistent disparities in neonatal outcomes across different population groups (4–8). These inequities are not only evident at birth but also have long-term consequences for those affected. In particular, McHale et al. (20) show that the negative influence of low socioeconomic status is especially pronounced for individuals born pre-term, leading to stronger adverse effects on cognitive and mental health outcomes. This evidence underscores the importance of examining how socioeconomic and ethnic factors intersect to shape disparities in pre-term birth outcomes, as addressed in our study.

There remains a gap in understanding how these inequities manifest within populations already at high risk, such as VP/VLBW individuals. Our study addresses this gap by focusing on the disparities in birth outcomes among VP/VLBW individuals, specifically examining the incidence of EP or ELBW.

Previous literature on inequities among those born VP has consistently shown a higher incidence of VP birth in deprived areas (21–23) and increased odds of VP birth among socio-economically disadvantaged groups (24). However, to our knowledge, no study has systematically quantified socioeconomic inequality in VP/VLBW populations using a comprehensive inequality index, such as the concentration index decomposition. This approach is particularly advantageous as it not only quantifies the magnitude of inequality but also decomposes the contribution of individual socioeconomic factors, allowing for a nuanced understanding of the drivers of inequality.

Our study further distinguishes itself by examining regional variations comparing health outcomes across multiple high-income countries, which provides a broader perspective on how socioeconomic factors influence disparities across diverse contexts. Unlike most previous studies that primarily focus on socioeconomic status (SES) and ethnicity, our analysis extends to a range of non-need variables, including parental education, maternal marital status, and infant sex.

Our results may appear counter-intuitive in light of previous findings in the literature. While many studies have documented that pre-term birth is generally pro-poor, more common among socioeconomically disadvantaged groups (6), we find that within the VP/VLBW sample, EP and ELBW may be pro-rich in some contexts. This contrast highlights the importance of assessing inequity within specific subgroups rather than across the entire population. One possible explanation is that our analysis is conditional on inclusion in the RECAP cohorts and therefore implicitly conditions on survival to cohort enrolment. Most contributing cohorts recruited survivors rather than all births, and we do not have complete data on neonatal deaths or stillbirths across all cohorts. Since neonatal survival is typically higher among more advantaged groups due to for example better access to neonatal intensive care (25, 26, 29), the observed concentration of EP and ELBW among the socioeconomically advantaged may partly reflect survival bias.

In our data, we do not observe neonatal mortality or stillbirths, so we cannot assess inequity in survival at birth; however, we do observe mortality before adulthood. Table A6 presents the inequity index in mortality before adulthood for countries with available data, showing a strongly pro-poor pattern—higher mortality among disadvantaged groups—which supports our underlying assumption. These findings underscore the need to consider post-natal selection mechanisms when interpreting patterns of inequity in high-risk populations. The cross-country differences observed in our analysis suggest that the relationship between socioeconomic background and birth outcomes is shaped not only by individual level characteristics but also by broader demographic and healthcare dynamics. In Norway and the Netherlands, we observe pro-rich inequity in EP and ELBW—seemingly counter to expectations given their strong welfare systems and universal access to care. However, this pattern likely reflects higher neonatal survival among socioeconomically advantaged groups in these countries, particularly for the most vulnerable infants.

Beyond survival bias, differences in the organization and regionalisation of perinatal care across countries may also contribute to the observed patterns. Variations in access to specialized neonatal services and in the timing of referral or in utero transfer for high-risk pregnancies may interact with socioeconomic status, potentially influencing survival and outcomes among VP/VLBW infants. Such system-level factors may therefore partly shape the cross-country differences in inequity observed in this study.

### 4.1 Strengths and limitations

The strength of the study lies in the use of harmonized, individual-level data from multiple high-income countries, covering cohorts of VP/VLBW individuals. This unique data structure allows for cross-country comparisons within a consistently defined high-risk population. While our analysis focused on birth outcomes, the persistence of inequity in this group is especially concerning given emerging evidence that socioeconomic disparities may become more pronounced as individuals age (27, 28). In terms of methodology, our use of the concentration index combined with the decomposition approach allowed us to distinguish between inequality arising from legitimate medical need and inequity driven by socioeconomic circumstances. This provides a nuanced picture of how different non-need factors contribute to disparities in birth outcomes, offering more policy-relevant insights than simple descriptive comparisons.

This study has some limitations. First, the selection criteria were not uniform across countries in the RECAP dataset. In particular, the UK and Canada cohorts included only individuals at the lower end of the GA distribution. This restriction leads to a truncated distribution of gestational age and birth weight in these cohorts, with limited variability in EP and ELBW. When outcomes exhibit limited variability, concentration indices and inequity measures are mechanically constrained and may be sensitive to small socioeconomic differences. As a result, both the magnitude and direction of inequity estimates in the UK and Canadian cohorts should be interpreted cautiously, as part of the cross-country variation may reflect cohort design rather than underlying structural differences in socioeconomic gradients. Second, we used parental education as our measure of socioeconomic position because consistent data on income and wealth were not available across all cohorts. While education is a widely accepted proxy, it does not capture all dimensions of socioeconomic advantage. In addition, the social and economic meaning of educational categories may differ across countries and historical periods, particularly given the varying birth years of the cohorts. Although we harmonized education using ISCED categories to improve comparability, residual cross-national and temporal differences in the returns to education may influence the observed gradients. Third, our analysis was limited by the availability of clinical need variables. In particular, data on maternal risky behaviors during pregnancy, such as smoking or substance use, were missing or inconsistently recorded in some cohorts, which may lead to an under-adjustment for relevant medical need in the inequity calculations. If important clinical risk factors are not fully captured, part of the observed socioeconomic gradient may reflect differences in underlying medical risk rather than unfair disparities, potentially leading to an overestimation of horizontal inequity. Fourth, some measures used in the NZ cohort, such as parental education and socioeconomic status (SES), were based on retrospective reporting in adulthood. This reliance on retrospective reports may introduce bias, as they could reflect inaccuracies in recalling the parents' actual status at the time of birth. Fifth, because the cohorts were born in different decades, secular improvements in neonatal intensive care and survival may have interacted with socioeconomic status over time, potentially influencing the observed pro-rich or pro-poor inequity patterns.

## 5 Conclusion

This multinational study reveals significant socioeconomic inequities in birth outcomes among very pre-term and very low birth weight infants across high-income countries. Our analysis demonstrates that the distribution of extreme pre-term birth and extremely low birth weight follows distinct socioeconomic gradients, with pro-rich inequities evident in the Netherlands and Norway contrasting with pro-poor patterns in Germany and New Zealand. These findings challenge conventional epidemiological understandings of pre-term birth disparities, as we observed the counterintuitive concentration of the most extreme adverse outcomes among socioeconomically advantaged groups in certain national contexts—a phenomenon potentially attributable to differential neonatal survival rates or variations in access to intensive care.

The decomposition of concentration indices identified parental education as the pre-dominant contributor to observed inequities in EP and ELBW, with additional significant effects from ethnicity and parental socioeconomic status. These disparities are particularly concerning given emerging evidence that early-life inequities among pre-term infants may amplify across the life course, leading to widening gaps in health and developmental outcomes during adulthood.

From a policy perspective, these findings underscore the necessity for dual-focused interventions addressing both clinical care pathways and social determinants of health. Targeted approaches should prioritize equitable access to high-quality care across the continuum of services, including timely and adequate antenatal care for high-risk pregnancies, equitable admission and treatment within neonatal intensive care units, and structured post-discharge follow-up and early developmental support for families of VP/VLBW infants, while simultaneously implementing family support programs that mitigate socioeconomic disadvantages. The persistence of these inequities into later life stages further emphasizes the importance of establishing longitudinal monitoring systems to track developmental trajectories and health outcomes among pre-term cohorts.

## Data Availability

The data analyzed in this study is subject to the following licenses/restrictions: the data supporting the results reported in this article are available upon request from the RECAP Pre-term Project and the individual data holders. Due to privacy restrictions, individual-level data cannot be shared publicly. For access to the data, researchers may contact the RECAP Pre-term Project at https://recap-pre-term.eu to request the minimal dataset necessary for replicating and building upon the findings reported in this study. Requests to access these datasets should be directed to https://recap-pre-term.eu.

## Footnotes

1 Project on Pre-term and Small for Gestational Age Infants.

2 Bavaria Longitudinal Study Cohort

3 Norwegian University of Science and Technology.

4
https://www.nichd.nih.gov/health/topics/preterm/conditioninfo/who_risk
