# Supplementary material for: Socioeconomic inequity in extreme outcomes within very pre-term and/or very low birthweight infants: evidence from multi-national cohorts
Source: Front Public Health. 2026 Apr 23;14:1791450. doi: 10.3389/fpubh.2026.1791450 (PMC13149178; doi:10.3389/fpubh.2026.1791450)
Supplement: Supplementary file 1 [file Supplementary_file_1.docx]

# Appendix

## Appendix: Van de Poel Method

The decomposition by van de Poel et al. (2012) refines the standard method by accounting for heterogeneity in the response to need across socioeconomic groups. This method includes:

- Homogeneous Contributions: The standard decomposition assuming uniform response to need.
- Corrected Need Effect: Adjusts for underestimation of legitimate variation in healthcare use due to heterogeneity.
- Heterogeneity Component: Captures inequity arising from differential responses to need among the poor.

The adjusted inequity measure is:

Inequity = CI - Homogeneous Need Contribution - Corrected Need Effect

$$C_{y}= \sum_{j} \gamma_{j}^{p}\frac{\bar{x_{j}}C_{xj}}{\bar{y}}+ \frac{2}{\bar{y}N}\sum_{j} (\gamma_{jgr}-\gamma_{j}^{p})\sum_{i} x_{ji}\left( R_{i}-\frac{1}{2} \right)+\frac{2}{\bar{y}N} \sum_{j} \sum_{i} x_{ji}\left( \gamma_{jg}-\gamma_{jgr} \right)(R_{i}-\frac{1}{2})+\sum_{k} \delta_{k}^{p}\frac{\bar{z_{k}}C_{zk}}{\bar{y}}+ \frac{2}{\bar{y}N} \sum_{k} \sum_{i} z_{ki}(\delta_{kg}-\delta_{k}^{p})(R_{i}-\frac{1}{2})+\frac{2}{\bar{y}} cov\left( \alpha_{g}, R_{i} \right)+\frac{2}{\bar{y}}cov\left( u_{i},R_{i} \right)$$

where:

- γⱼᴾ and δₖᴾ are the parameters of need and non-need variables, respectively, in the pooled sample.
- γⱼg and δₖg are the parameters of the need and non-need variables, respectively, in each income group.
- γⱼgʳ is the parameter of the need variable for the reference group (typically the wealthiest subgroup with the best medical access).
- The first and second term are homogeneous need and corrected need effect, respectively.

By applying this approach, van de Poel et al. (2012) demonstrate that traditional models often underestimate pro-rich inequity, as they fail to account for how healthcare responsiveness varies among the poor.

## Extra figures

Figure A1: concentration index and inequity index for birth outcomes: GA and BW


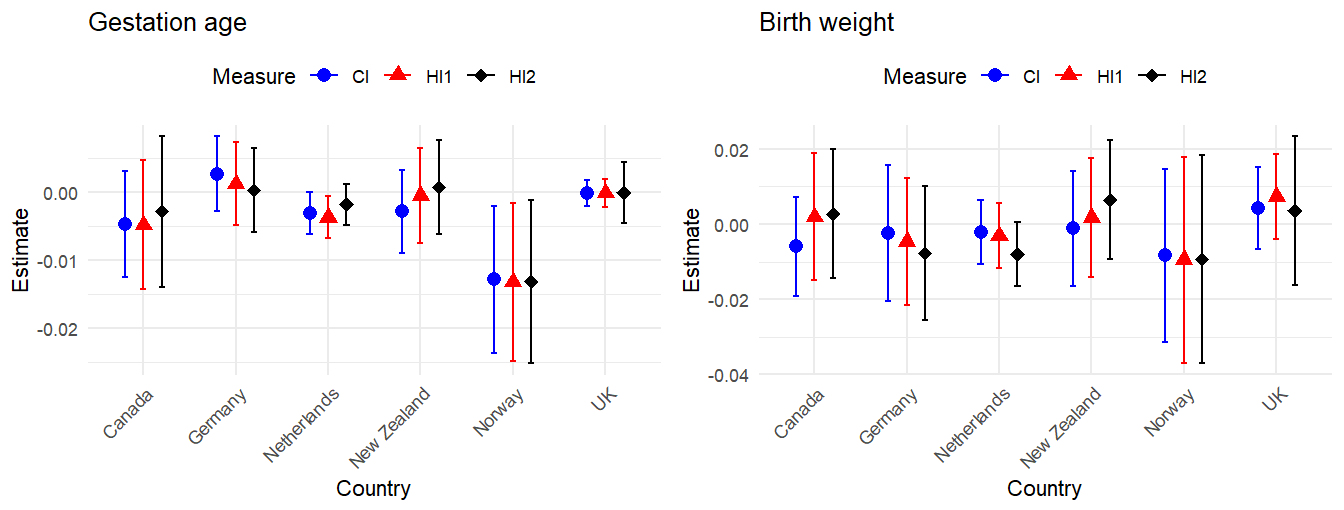


(a) (b)

Note: CI is concentration index. HI1 is the horizontal inequity index based on conventional method and HI2 is the horizontal inequity index based on [Van de Poel et al.](#_bookmark30) ([2012](#_bookmark30))

Figure A2: Contribution of non-need variables to the inequality in GA and BW


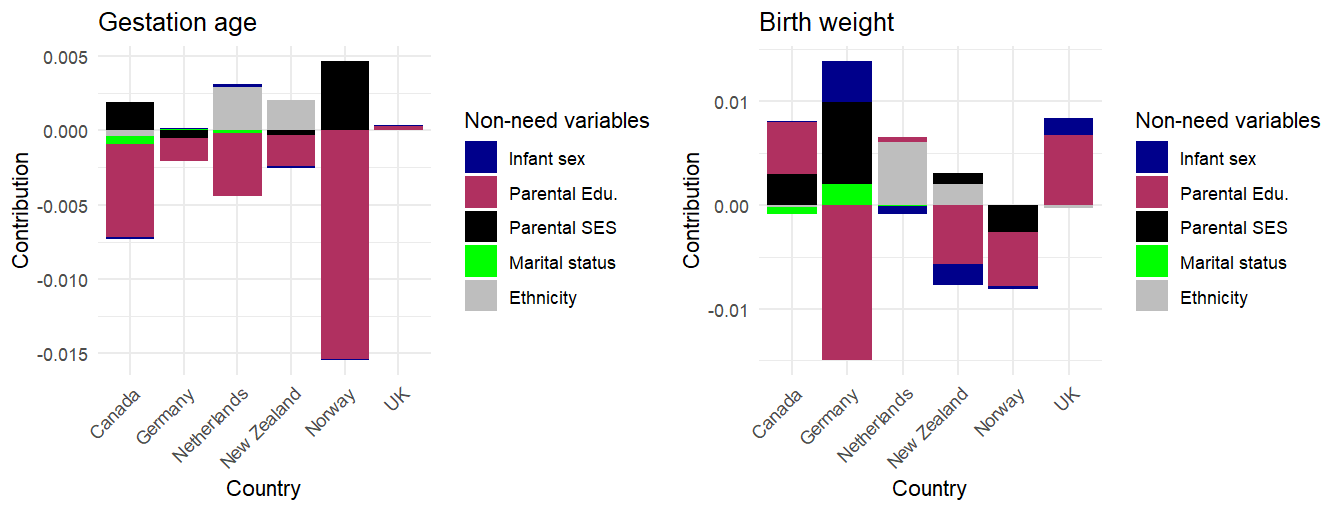


(a) (b)

## Extra tables

Table A1: Summary Statistics of outcome, need and non-need variables

|  | variable | N | mean | sd | CI | CI sd | CI pval |
| --- | --- | --- | --- | --- | --- | --- | --- |
| **Canada** | GA | 179 | 26.994 | 2.255 | -0.005 | 0.004 | 0.230 |
|  | BW | 179 | 837.374 | 122.935 | -0.006 | 0.007 | 0.372 |
|  | EP | 179 | 0.682 | 0.467 | 0.069 | 0.100 | 0.493 |

| ELBW | 179 | 0.950 | 0.219 | 0.151 | 0.196 | 0.441 |
| --- | --- | --- | --- | --- | --- | --- |
| Parental education (low) | 154 | 0.214 | 0.412 | -1 | 0.042 | 0 |
| Parental education (middle) | 154 | 0.623 | 0.486 | 0.138 | 0.131 | 0.292 |
| Parental education (high) | 154 | 0.162 | 0.370 | 1 | 0.035 | 0 |
| Maternal age | 178 | 25.972 | 5.657 | 0.053 | 0.009 | 0 |
| Maternal smoking | 119 | 0.345 | 0.477 | -0.110 | 0.115 | 0.338 |
| Nulliparous | 179 | 0.296 | 0.458 | 0.049 | 0.098 | 0.620 |
| Maternal marital status (single=1) | 179 | 0.128 | 0.336 | -0.463 | 0.117 | 0.0001 |
| Ethnicity (Caucasian=1) | 179 | 0.916 | 0.278 | -0.022 | 0.186 | 0.905 |
| Infant sex (female=1) | 179 | 0.469 | 0.500 | 0.013 | 0.094 | 0.894 |
| Parental SES | 172 | 2.430 | 1.214 | 0.107 | 0.021 | 0.00000 |
| **Germany** |  |  |  |  |  |  |
| GA | 444 | 29.356 | 2.852 | 0.003 | 0.003 | 0.338 |
| BW | 444 | 1,180.216 | 366.450 | -0.002 | 0.009 | 0.801 |
| EP | 444 | 0.270 | 0.445 | -0.026 | 0.131 | 0.844 |
| ELBW | 444 | 0.345 | 0.476 | -0.094 | 0.098 | 0.341 |
| Parental education (low) | 280 | 0.036 | 0.186 | -1 | 0.012 | 0 |
| Parental education (middle) | 280 | 0.746 | 0.436 | -0.718 | 0.111 | 0 |
| Parental education (high) | 280 | 0.218 | 0.414 | 1 | 0.032 | 0 |
| Maternal age | 444 | 28.563 | 5.004 | 0.019 | 0.006 | 0.001 |
| Nulliparous | 415 | 0.528 | 0.500 | 0.038 | 0.069 | 0.579 |
| Maternal marital status (single=1) | 283 | 0.081 | 0.274 | -0.227 | 0.138 | 0.102 |
| Infant sex (female=1) | 444 | 0.464 | 0.499 | -0.085 | 0.069 | 0.221 |
| Parental SES | 287 | 1.864 | 0.723 | 0.126 | 0.012 | 0 |
| **Netherlands** |  |  |  |  |  |  |
| GA | 1,336 | 30.319 | 2.863 | -0.003 | 0.002 | 0.048 |
| BW | 1,336 | 1,248.962 | 319.114 | -0.002 | 0.004 | 0.619 |
| EP | 1,336 | 0.192 | 0.394 | 0.096 | 0.054 | 0.078 |
| ELBW | 1,336 | 0.219 | 0.413 | 0.040 | 0.048 | 0.404 |
| Parental education (low) | 1,019 | 0.388 | 0.487 | -1 | 0.025 | 0 |
| Parental education (middle) | 1,019 | 0.362 | 0.481 | 0.215 | 0.039 | 0.00000 |
| Parental education (high) | 1,019 | 0.250 | 0.433 | 1 | 0.018 | 0 |
| Maternal age | 1,305 | 27.285 | 4.868 | 0.028 | 0.003 | 0 |
| Maternal smoking | 1,171 | 0.331 | 0.471 | -0.190 | 0.039 | 0.00000 |
| Nulliparous | 1,333 | 0.520 | 0.500 | -0.010 | 0.037 | 0.788 |
| Maternal marital status (single=1) | 1,330 | 0.123 | 0.328 | -0.127 | 0.062 | 0.041 |
| Ethnicity (Caucasian=1) | 1,326 | 0.857 | 0.350 | 0.185 | 0.057 | 0.001 |
| Infant sex (female=1) | 1,332 | 0.470 | 0.499 | 0.033 | 0.036 | 0.369 |
| **New Zealand** |  |  |  |  |  |  |
| GA | 413 | 28.886 | 2.588 | -0.003 | 0.003 | 0.357 |
| BW | 413 | 1,117.412 | 244.694 | -0.001 | 0.008 | 0.886 |
| EP | 413 | 0.305 | 0.461 | 0.049 | 0.084 | 0.555 |
| ELBW | 413 | 0.320 | 0.467 | 0.096 | 0.078 | 0.217 |
| Parental education (low) | 250 | 0.212 | 0.410 | -1 | 0.033 | 0 |
| Parental education (middle) | 250 | 0.268 | 0.444 | -0.421 | 0.069 | 0 |
| Parental education (high) | 250 | 0.520 | 0.501 | 1 | 0.066 | 0 |

| Maternal age | 370 | 25.924 | 5.612 | 0.032 | 0.007 | 0.00001 |
| --- | --- | --- | --- | --- | --- | --- |
| Nulliparous | 249 | 0.482 | 0.501 | -0.071 | 0.073 | 0.336 |
| Ethnicity (Caucasian=1) | 250 | 0.696 | 0.461 | 0.284 | 0.090 | 0.002 |
| Infant sex | 413 | 0.494 | 0.501 | 0.059 | 0.074 | 0.426 |
| Parental SES | 250 | 48.416 | 17.979 | 0.104 | 0.011 | 0 |
| **Norway** |  |  |  |  |  |  |
| GA | 121 | 28.331 | 2.564 | -0.013 | 0.005 | 0.019 |
| BW | 121 | 1,121.653 | 260.801 | -0.008 | 0.012 | 0.480 |
| EP | 121 | 0.380 | 0.487 | 0.219 | 0.163 | 0.179 |
| ELBW | 121 | 0.306 | 0.463 | 0.135 | 0.165 | 0.413 |
| Parental education (low) | 71 | 0.014 | 0.119 | -1 | 0.014 | 0 |
| Parental education (middle) | 71 | 0.563 | 0.499 | -0.935 | 0.141 | 0 |
| Parental education (high) | 71 | 0.423 | 0.497 | 1 | 0.102 | 0 |
| Maternal age | 119 | 27.378 | 5.006 | 0.008 | 0.011 | 0.438 |
| Infant sex (female=1) | 121 | 0.430 | 0.497 | -0.006 | 0.138 | 0.963 |
| Parental SES | 72 | 3.292 | 1.250 | 0.140 | 0.018 | 0 |
| **UK** |  |  |  |  |  |  |
| GA | 315 | 24.492 | 0.679 | -0.0001 | 0.001 | 0.898 |
| BW | 315 | 747.289 | 114.418 | 0.004 | 0.006 | 0.436 |
| EP | 315 | 1 | 0 | 0 |  |  |
| ELBW | 315 | 0.990 | 0.097 | -0.459 | 0.447 | 0.304 |
| Parental education (low) | 263 | 0.236 | 0.425 | -1 | 0.034 | 0 |
| Parental education (middle) | 263 | 0.703 | 0.458 | 0.590 | 0.107 | 0.00000 |
| Parental education (high) | 263 | 0.061 | 0.239 | 1 | 0.016 | 0 |
| Maternal age | 313 | 28.460 | 5.903 | 0.012 | 0.008 | 0.119 |
| Nulliparous | 314 | 0.506 | 0.501 | 0.072 | 0.072 | 0.318 |
| Ethnicity (Caucasian=1) | 306 | 0.788 | 0.410 | -0.075 | 0.094 | 0.425 |
| Infant sex (female=1) | 315 | 0.508 | 0.501 | -0.054 | 0.071 | 0.446 |

*Note:* Infant sex is a binary variable which takes one when the child is female and zero otherwise. Paren education has three levels. Low educated refers to lower secondary education (ISCED 0-2), mid educat corresponds to upper secondary and post-secondary non-tertiary education (ISCED 3-5), and high educat encompasses tertiary education (ISCED 6-8). Parental SES is a continuous index of socioeconomic stat Marital status is a binary variable which takes one when the mother is not married and not in civil partnersh and zero otherwise. Ethnicity is a binary variable which takes one when the mother is Caucasian and ze otherwise.

Table A2: CI, HI1 and HI2 and associated p-values for countries of the study for four outcome variables: GA, BW, EP and ELBW

Country CI p value HI1 p value HI2 p value

**Gestation age**

| Canada | -0*.*005 | 0*.*230 | -0*.*005 | 0*.*316 | -0*.*003 | 0*.*609 |
| --- | --- | --- | --- | --- | --- | --- |
| Germany | 0*.*003 | 0*.*338 | 0*.*001 | 0*.*702 | 0*.*0002 | 0*.*949 |
| Netherlands | -0*.*003 | 0*.*048 | -0*.*004 | 0*.*017 | -0*.*002 | 0*.*227 |
| New Zealand | -0*.*003 | 0*.*357 | -0*.*001 | 0*.*875 | 0*.*001 | 0*.*850 |
| Norway | -0*.*013 | 0*.*019 | -0*.*013 | 0*.*025 | -0*.*013 | 0*.*031 |
| UK | -0*.*0001 | 0*.*898 | -0*.*0002 | 0*.*875 | -0*.*0001 | 0*.*961 |
| **Birth weight** | | | | | | |
| Canada | -0*.*006 | 0*.*372 | 0*.*002 | 0*.*820 | 0*.*003 | 0*.*752 |
| Germany | -0*.*002 | 0*.*801 | -0*.*005 | 0*.*593 | -0*.*008 | 0*.*395 |
| Netherlands | -0*.*002 | 0*.*619 | -0*.*003 | 0*.*474 | -0*.*008 | 0*.*069 |
| New Zealand | -0*.*001 | 0*.*886 | 0*.*002 | 0*.*831 | 0*.*006 | 0*.*425 |
| Norway | -0*.*008 | 0*.*480 | -0*.*009 | 0*.*499 | -0*.*009 | 0*.*507 |
| UK | 0*.*004 | 0*.*436 | 0*.*007 | 0*.*202 | 0*.*004 | 0*.*721 |
| **Extreme preterm** | | | | | | |
| Germany | -0*.*026 | 0*.*844 | 0*.*0004 | 0*.*998 | 0*.*001 | 0*.*989 |
| Netherlands | 0*.*096 | 0*.*078 | 0*.*120 | 0*.*046 | 0*.*171 | 0 |
| New Zealand | 0*.*049 | 0*.*555 | 0*.*029 | 0*.*746 | -0*.*020 | 0*.*644 |
| Norway | 0*.*219 | 0*.*179 | 0*.*219 | 0*.*141 | 0*.*210 | 0*.*019 |
| **Extreme low birth weight** | | | | | | |
| Germany | -0*.*094 | 0*.*341 | -0*.*078 | 0*.*446 | -0*.*046 | 0*.*380 |
| Netherlands | 0*.*040 | 0*.*404 | 0*.*074 | 0*.*165 | 0*.*101 | 0*.*00003 |
| New Zealand | 0*.*096 | 0*.*217 | 0*.*081 | 0*.*314 | -0*.*001 | 0*.*982 |
| Norway | 0*.*135 | 0*.*413 | 0*.*148 | 0*.*407 | 0*.*143 | 0*.*233 |

*Note:* CI is concentration index. HI1 is the horizontal inequity index based on conventional method and HI2 is the horizontal inequity index based on [Van de Poel et al.](#_bookmark30) ([2012](#_bookmark30)) method Pooled results for all countries

Table A3: Contributions of non-need variables to inequity

Country Ethnicity Infant sex Marital status Parental Edu. Parental SES

**GA**

| Canada | -0.0004 | -0.0001 | -0.0010 | -0.0060 | 0.0020 |
| --- | --- | --- | --- | --- | --- |
| Germany | N/A | 0.0001 | 0.0001 | -0.0010 | -0.0010 |
| Netherlands | 0.0030 | 0.0002 | -0.0002 | -0.0040 | N/A |
| New Zealand | 0.0020 | -0.0001 | N/A | -0.0020 | -0.0003 |
| Norway | N/A | -0.0000 | N/A | -0.0150 | 0.0050 |
| UK | *<*-0.0001 | 0.0001 | N/A | 0.0002 | N/A |
| **Birth weight** |  |  |  |  |  |
| Canada | -0.0002 | 0.0001 | -0.0010 | 0.0050 | 0.0030 |
| Germany | N/A | 0.0040 | 0.0020 | -0.0150 | 0.0080 |
| Netherlands | 0.0060 | -0.0010 | -0.0001 | 0.0005 | N/A |
| New Zealand | 0.0020 | -0.0020 | N/A | -0.0060 | 0.0010 |
| Norway | N/A | -0.0002 | N/A | -0.0050 | -0.0030 |
| UK | -0.0003 | 0.0020 | N/A | 0.0070 | N/A |
| **EP** |  |  |  |  |  |
| Germany | N/A | -0.0070 | -0.0010 | 0.0560 | -0.0380 |
| Netherlands | -0.0380 | -0.0030 | -0.0020 | 0.0440 | N/A |
| New Zealand | -0.0470 | 0.0020 | N/A | 0.0020 | 0.0150 |
| Norway | N/A | 0.0000 | N/A | 0.0710 | -0.0190 |
| **ELBW** |  |  |  |  |  |
| Germany | N/A | -0.0070 | -0.0120 | 0.0100 | -0.0020 |
| Netherlands | -0.0680 | 0.0010 | 0.0050 | 0.0530 | N/A |
| New Zealand | -0.0240 | 0.0040 | N/A | 0.0290 | 0.0090 |
| Norway | N/A | 0.0001 | N/A | 0.0770 | 0.0160 |
|  |  |  |  |  |  |

Table A4: CI, HI1 and HI2 and associate p-values for the pooled data

| Outcome | CI Estimate | p-value CI | HI1 Estimate | p-value HI1 | HI2 Estimate | p-value HI2 |
| --- | --- | --- | --- | --- | --- | --- |
| GA | -0.014 | <0.001 | -0.014 | <0.001 | -0.014 | <0.001 |
| BW | -0.03 | <0.001 | -0.03 | <0.001 | -0.031 | <0.001 |
| EP | 0.243 | <0.001 | 0.239 | <0.001 | 0.245 | <0.001 |
| ELBW | 0.199 | <0.001 | 0.199 | <0.001 | 0.201 | <0.001 |

*Note:* CI is concentration index. HI1 is the horizontal inequity index based on conventional method and HI2 is the horizontal inequity index based on [Van de Poel et al.](#_bookmark30) ([2012](#_bookmark30)) method. Pooled results are for all six countries

Table A5: Contribution of non-need variables in the pooled data

| Measure | Infant sex | parental edu. |
| --- | --- | --- |
| GA | *<*-0.001 | -0.002 |
| BW | -0.001 | 0.001 |
| EP | 0.002 | -0.011 |
| ELBW | 0.002 | -0.010 |

Table A6: Inequality and inequity indices for mortality prior to adulthood

| Country | CI Estimate | p-value CI | HI1 Estimate | p-value HI1 | HI2 Estimate | p-value HI2 |
| --- | --- | --- | --- | --- | --- | --- |
| Canada | -0.252 | 0.197 | -0.252 | 0.597 | -0.465 | 0.915 |
| Germany | -0.124 | 0.365 | -0.117 | 0.377 | -0.097 | 0.209 |
| Netherlands | 0.001 | 0.987 | -0.004 | 0.937 | -0.009 | 0.726 |

*Note:* CI is concentration index. HI1 is the horizontal inequity index based on conventional method and HI2 is the horizontal inequity index based on [Van de Poel et al.](#_bookmark30) ([2012](#_bookmark30)) method Pooled results for all countries
